# Design and Interaction Control of a New Bilateral Upper-Limb Rehabilitation Device

**DOI:** 10.1155/2017/7640325

**Published:** 2017-09-20

**Authors:** Qing Miao, Mingming Zhang, Yupu Wang, Sheng Q. Xie

**Affiliations:** ^1^School of Mechanical and Electrical Engineering, Wuhan University of Technology, Wuhan, China; ^2^Department of Mechanical Engineering, The University of Auckland, Auckland, New Zealand; ^3^Medical & Rehabilitation Equipment Research Centre, Tongji Zhejiang College, Jiaxing, China

## Abstract

This paper proposed a bilateral upper-limb rehabilitation device (BULReD) with two degrees of freedom (DOFs). The BULReD is portable for both hospital and home environment, easy to use for therapists and patients, and safer with respect to upper-limb robotic exoskeletons. It was implemented to be able to conduct both passive and interactive training, based on system kinematics and dynamics, as well as the identification of real-time movement intention of human users. Preliminary results demonstrate the potential of the BULReD for clinical applications, with satisfactory position and interaction force tracking performance. Future work will focus on the clinical evaluation of the BULReD on a large sample of poststroke patients.

## 1. Introduction

In the United States, more than 700,000 people suffer from stroke each year, and approximately two-thirds of these individuals survive and require rehabilitation [1]. In New Zealand (NZ), there are an estimated 60,000 stroke survivors, and many of them have mobility impairments [2]. Stroke is the third reason for health loss and takes the proportion of 3.9 percent, especially for the group starting on middle age, suffering the stroke as a nonfatal disease in NZ [3]. Professor Caplan who studies Neurology at Harvard Medical School describes stroke as a term which is a kind of brain impairment as a result of abnormal blood supply in a portion of the brain [4]. The brain injury is most likely leading to dysfunctions and disabilities. These survivors normally have difficulties in activities of daily living, such as walking, speaking, and understanding, and paralysis or numbness of the human limbs. The goals of rehabilitation are to help survivors become as independent as possible and to attain the best possible quality of life.

Physical therapy is conventionally delivered by the therapist. While this has been demonstrated as an effective way for motor rehabilitation [5], it is time-consuming and costly. Treatments manually provided by therapists require to take place in a specific environment (in a hospital or rehabilitation center) and may last several months for enhanced rehabilitation efficacy [6]. A study by Kleim et al. [7] has shown that physical therapy like regular exercises can improve plasticity of a nervous system and then benefits motor enrichment procedures in promoting rehabilitation of brain functional models. It is a truth that physical therapy should be a preferable way to take patients into regular exercises and guided by a physical therapist, but Chang et al. [8] showed that it is a money-consuming scheme. Robot-assisted rehabilitation solutions, as therapeutic adjuncts to facilitate clinical practice, have been actively researched in the past few decades and provide an overdue transformation of the rehabilitation center from labor-intensive operations to technology-assisted operations [9]. The robot could also provide a rich stream of data from built-in sensors to facilitate patient diagnosis, customization of the therapy, and maintenance of patient records. As a popular neurorehabilitation technique, Liao et al. [10] indicated that robot-assisted therapy presents market potential due to quantification and individuation in the therapy session. The quantification of robot-assisted therapy refers that a robot can provide consistent training pattern without fatigue with the given parameter. The characterization of individuation allows therapists to customize a specific training scheme for an individual.

Many robotic devices have been developed in recent years for stroke rehabilitation and show great potential for clinical applications [11, 12]. Typical upper-limb rehabilitation devices are MIME, MIT-Manus, ARM Guide, NeReBot, and ARMin [5, 13–21]. Relevant evidences demonstrated that these robots are effective for upper-limb rehabilitation but mostly for the one side of the human body. Further, upper-limb rehabilitation devices can be unilateral or bilateral [22–24]. Despite the argument between these two design strategies, bilateral activities are more common than unilateral activities in daily living. Liu et al. [25] pointed that the central nervous system dominates the human movement with coordinating bilateral limb to act in one unit instead of independent unilateral actions. From this point, bilateral robots are expected to be more potential than unilateral devices. Robotic devices for upper-limb rehabilitation can be also divided into two categories in terms of structure: the exoskeleton and the end-effector device [26]. Two examples of upper-limb exoskeletons are the arm exoskeleton [27] and the RUPERT IV [28]. In addition, Lum et al. [13] incorporated a PUMA 560 robot (Staubli Unimation Inc., Duncan, South Carolina) to apply forces to the paretic limbs in the MIME system. This robotic device can be made for both unilateral and bilateral movements in a three-dimensional space. To summarize, existing robotic exoskeletons for upper-limb rehabilitation are mostly for unilateral training.

There are some devices that have been specially designed for bilateral upper-limb training for poststroke rehabilitation. van Delden et al. [29] conducted a systematic review to provide an overview and qualitative evaluation of the clinical applications of bilateral upper-limb training devices. A systematic search found a total of six mechanical devices and 14 robotic bilateral upper-limb training devices, with a comparative analysis in terms of mechanical and electromechanical characteristics, movement patterns, targeted part, and active involvement of the upper limb, training protocols, outcomes of clinical trials, and commercial availability. Obviously, these mechanical devices require the human limbs to actively move for training, while the robotic ones can be operated in both passive and active modes. However, few of these robotic bilateral upper-limb training devices have been commercially available with current technology. For example, the exoskeleton presented in [30] requires the development of higher power-to-weight motors and structural materials to make it mobile and more compact.

The University of Auckland developed an end-effector ReachHab device to assist bilateral upper-limb functional recovery [31]. However, this device suffered from some limitations, such as deformation of the frame leading to significant vibration, also hard to achieve satisfactory control performance. This paper presents the design and interaction control of an improved bilateral upper-limb rehabilitation device (BULReD). This device is portable for both hospital and home environment, easy to use for therapists and patients, and safer with respect to upper-limb robotic exoskeletons. This paper is organized as follows. Following Introduction, a detailed description of the BULReD is given, including mechanical design, electrical design, kinematics, and dynamics. Then, the control design is presented for both passive training and interactive training, as well as the fuzzy-based adaptive training. Experiments and Results is introduced next and the last is Conclusion.

## 2. Bilateral Upper-Limb Rehabilitation Device (BULReD)

### 2.1. Mechanical Design

**Figure 1:**
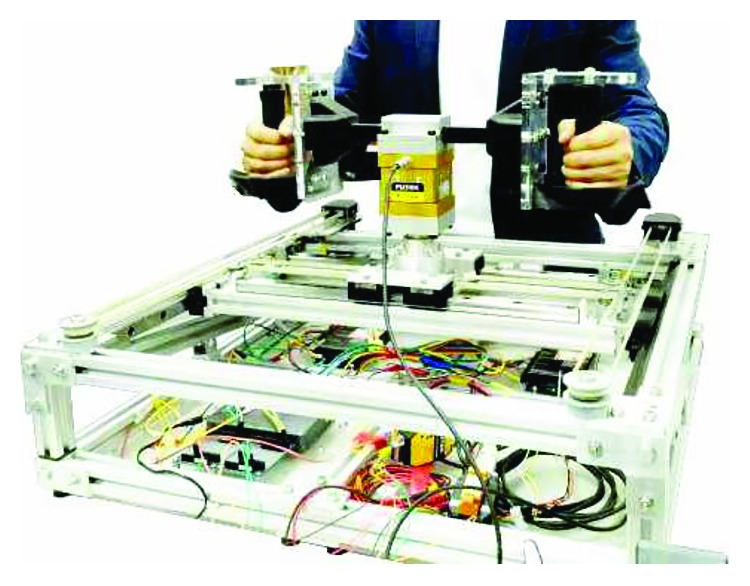
The BULReD on a lab table.

The BULReD aims to deliver bilateral upper-limb exercises to stroke patients. Overview of the BULReD is shown in Figure 1. This device has two DOFs for the planar exercises of human upper limbs.

**Figure 2:**
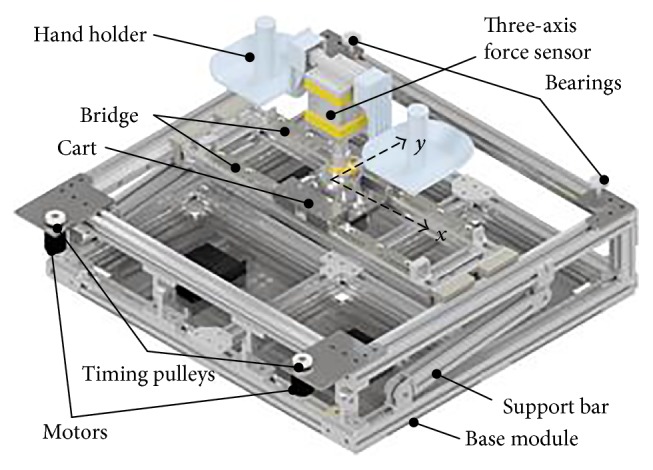
A three-dimensional model of the BULReD designed in Solidwork.

To make the structural design clear to the readers, a three-dimensional model design in Solidwork is also given in Figure 2. The BULReD mainly consists of three components (the base module, the motion module, and the hand holder). The base module acts as a foundation to support the whole motion module and also a container for some electronic components. There is a support bar in the base module, which can be used to adjust the angle of inclination of the BULReD. The motion module consists of two mutually perpendicular linear slide systems, the bridge and the cart. The linear slide systems are used to transfer the rotatory motion of motors to the linear motion of the sliders by using two timing belts. The bridge moves along the *y*-axis rail, and the cart is along the *x*-axis rail. The hand holder is rigidly connected with the cart through a three-axis force sensor. Some bearings are also set to allow low friction motion transmission.

The inclination angle of the BULReD is considered to be 0° when the motion module is parallel to the bottom base. By moving the attachment position of the support bar, the angle of inclination can be adjusted up to 60°. Such a design can make patients have a larger workspace for upper-limb rehabilitation exercises. The hand holder is designed based on anthropometry. Kaya et al. [32] suggested that the shoulder breadth has the mean measurement of 355.8 mm (female) and 389.4 mm (male) at age 17. In the prototype of the BULReD, the designed shoulder breadth is set at 38 cm. Fransson and Winkel [33] indicated that hand size measurements have the maximum finger length and handbreadth between women's and men's mean values of 72 mm and 90 mm, respectively. In the prototype, the handle height is set at 90 mm. However, these two parameters can be easily made to be adjustable to allow the use on patients with different sizes.

### 2.2. Electrical Design

The electrical component of the BULReD consists of two Maxon DC motors (each has one brushless motor and one gearbox), two motor controllers (Maxon ESCON 50/5), a three-axis force sensor (FUTEK MTA400), three amplifiers (FUTEK CSG110), and an embedded controller (NI myRIO-1900). Predefined data and those from the motor controller communicate with the embedded controller through digital input/output (DI/O), analog input (AI), and analog output (AO). Specifically, the myRIO outputs signals to drive servo motors and obtains feedback inputs of the position. The servo system provides speed feedback based on the built-in encoder with 2048 counts per turn. The position feedback is implemented by the computation of the speed feedback. The motor controller provides a stable power supply for DC motor and internally realizes closed loop speed control. The three-axis force sensor is used to measure real-time human-robot interaction to facilitate the implementation of the interactive training. For training safety, four limit switches are also set up, located at the corner of the workbench. They are also used as a reference to set the starting point.

### 2.3. Kinematics

**Figure 3:**
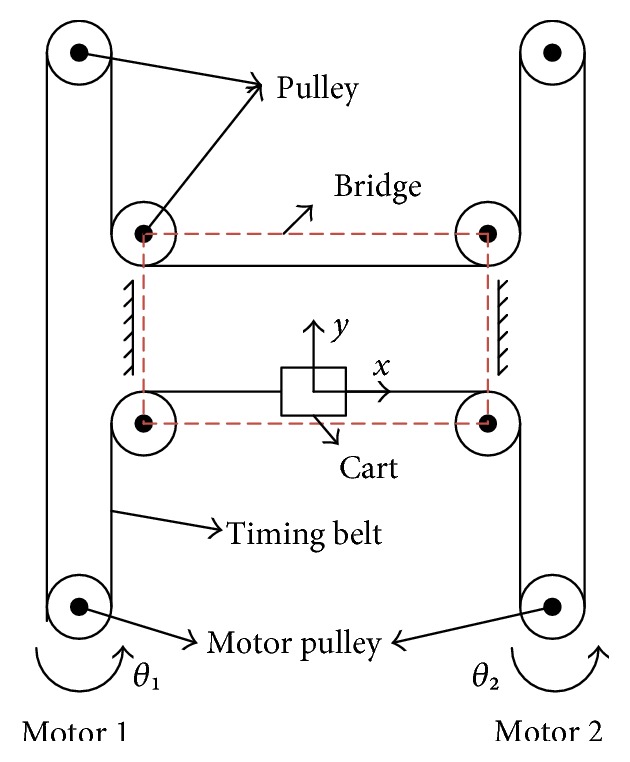
The schematic mechanism of the BULReD.

H-Bot (also known as H-gantry or H-frame) is a kind of XY positioning system which is commonly used in industries. It can place a part or carry a tool in a planar space. A model of the H-Bot consists of two mutually perpendicular linear slide systems and connects with the power source by timing belts [34]. It is driven by two independent motors in the cooperative work and results in planar motions.  Figure 3 presents the schematic mechanism of the BULReD, where the bridge moves along the *y*-axis and the cart moves along the *x*-axis. The hand holder is rigidly connected with the cart and forms a planar motion area.

The planar displacement of the cart is obtained from two rotational motors (motor 1 and motor 2 in Figure 3). Forward kinematics can be used to derive the linear displacement Δ*X* and Δ*Y* of the cart from two angular displacements Δ*θ*_1_ and Δ*θ*_2_ of the motors. The forward kinematic model is described in (1), where [Δ*X* Δ*Y*]^*T*^ denotes the position and orientation vector, ${\left[\begin{array}{cc} \Delta \theta_{1} & \Delta \theta_{2} \end{array}\right]}^{T}\;$is the angular displacement vector, and *A* is the initial forward kinematic matrix.

(1) $$\begin{array}{c} \left[\begin{array}{c} \Delta X\\ \Delta Y \end{array}\right]=A\;\cdot \;\left[\begin{array}{c} \Delta \theta_{1}\\ \Delta \theta_{2} \end{array}\right]=\left[\begin{array}{cc} a_{11} & a_{12}\\ a_{21} & a_{22} \end{array}\right]\cdot \left[\begin{array}{c} \Delta \theta_{1}\\ \Delta \theta_{2} \end{array}\right]. \end{array}$$

Table 1 is presented to give readers a qualitative description of the motion transmission mechanism on the BULReD. For example, the positive displacement along the *x*-axis can be achieved by two motors in an anticlockwise rotation (motion pattern 2 in Table 1), and that of the *y*-axis can be obtained by motor 1 in an anticlockwise rotation and motor 2 in a clockwise rotation (motion pattern 3 in Table 1).

The forward kinematic matrix is finally given in (2), where *r* is the radius of the motor pulley. By combining (1) and (2), the forward kinematic model is obtained in (3). The inverse kinematic model can be obtained by MATLAB matrix inverse function and is shown in (4) and (5), where *γ* is the reduction ratio of the gearhead, *U*_*i*_ is the control voltage of the motor, and *k*_*t*_ is the speed constant of the motor.

(2) $$\begin{array}{c} a_{11}=a_{12}=a_{21}=\frac{1}{2}r,a_{22}=-\frac{1}{2}r, \end{array}$$

(3) $$\begin{array}{c} \left[\begin{array}{c} \Delta X\\ \Delta Y \end{array}\right]=\left[\begin{array}{cc} \frac{1}{2}r & \frac{1}{2}r\\ \frac{1}{2}r & -\frac{1}{2}r \end{array}\right]\cdot \left[\begin{array}{c} \Delta \theta_{1}\\ \Delta \theta_{2} \end{array}\right], \end{array}$$

(4) $$\begin{array}{c} \left[\begin{array}{c} \Delta \theta_{1}\\ \Delta \theta_{2} \end{array}\right]=\left[\begin{array}{cc} \frac{1}{r} & \frac{1}{r}\\ \frac{1}{r} & -\frac{1}{r} \end{array}\right]\cdot \left[\begin{array}{c} \Delta X\\ \Delta Y \end{array}\right], \end{array}$$

(5) $$\begin{array}{c} \Delta \theta_{i}=\frac{2\pi }{60}\gamma^{-1}\;\cdot \;U_{i}\;\cdot \;k_{t}\;\cdot \;t\;\left(i=1,2\right). \end{array}$$

### 2.4. Dynamics

**Figure 4:**
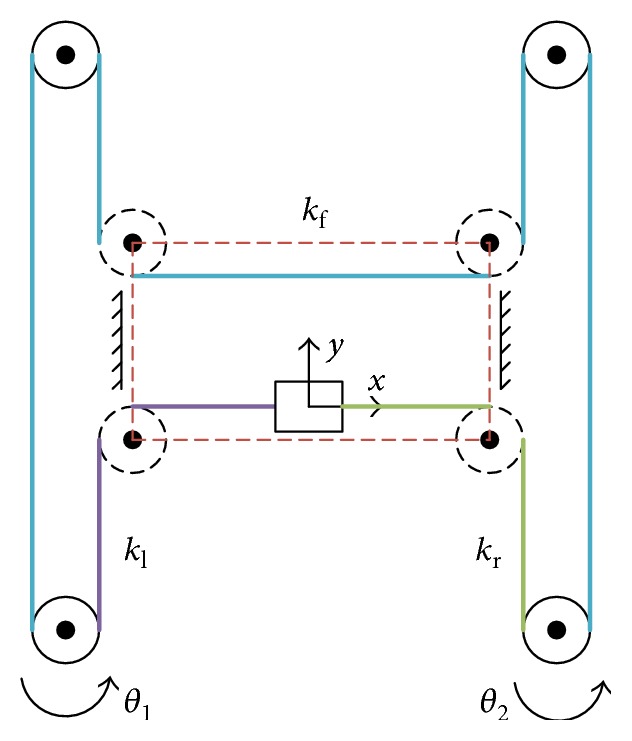
The simplified dynamic model of the BULReD (*k*_f_, *k*_l_, and  *k*_r_ represent the stiffness of the forward section, the left section, and the right section of the motion module, resp.).

A simplified dynamic model of the BULReD was developed with fewer parameters and will be used in the control system. In the BULReD, there are two timing pulleys with the same size, and others are low-friction bearings. Since the H-Bot system intends to transfer rotary movement of the two motors with two timing pulleys into linear movement, for simplification, the used bearings can be considered to be frictionless and cause no effects on the movement of the bridge and the cart. The timing pulleys are also considered to be frictionless to reduce the computational burden. A diagram is shown in Figure 4(), where the motion module is divided into three sections with different colors, the left section (purple), the right section (green), and the forward section (blue).

The dynamic energy of each section in the simplified system can be written as

(6) $$\begin{array}{c} T_{x}=\frac{1}{2}m_{cart}{\dot{x}}^{2}, \end{array}$$

(7) $$\begin{array}{c} T_{y}=\frac{1}{2}m_{bridge}{\dot{y}}^{2}, \end{array}$$

(8) $$\begin{array}{c} T_{i}=\frac{1}{2}J_{i}\dot{\theta_{i}^{2}}\;\left(i=1,2\right). \end{array}$$

The potential energy of the simplified system is stored in the three sections and shown in the following:

(9) $$\begin{array}{c} V_{f}=\frac{1}{2}k_{f}{\left(\theta_{1}r-\theta_{2}r-2y\right)}^{2}, \end{array}$$

(10) $$\begin{array}{c} V_{l}=\frac{1}{2}k_{l}{\left(-\theta_{1}r+x+y\right)}^{2}, \end{array}$$

(11) $$\begin{array}{c} V_{r}=\frac{1}{2}k_{r}{\left(\theta_{2}r-x+y\right)}^{2}. \end{array}$$

The Lagrange equation is applied to build the dynamic model and is shown in (12). Combining the virtual work theory as well, (13) is obtained.

(12) $$\begin{array}{c} L=\frac{1}{2}m_{cart}{\dot{x}}^{2}+\frac{1}{2}m_{bridge}{\dot{y}}^{2}+\frac{1}{2}J_{1}\dot{\theta_{1}^{2}}+\frac{1}{2}J_{2}\dot{\theta_{2}^{2}}-\frac{1}{2}k_{f}{\left(\theta_{1}r-\theta_{2}r-2y\right)}^{2}-\frac{1}{2}k_{l}{\left(-\theta_{1}r+x+y\right)}^{2}-\frac{1}{2}k_{r}{\left(\theta_{2}r-x+y\right)}^{2}, \end{array}$$

(13) $$\begin{array}{c} \delta W^{NC}=-c_{cart}\dot{x}\delta x-c_{bridge}\dot{y}\delta y+\left(\tau_{1}-c_{1}\dot{\theta_{1}}\right)\delta \theta_{1}+\left(\tau_{2}-c_{2}\dot{\theta_{2}}\right)\delta \theta_{2}. \end{array}$$

Substituting Lagrange's equation given, the simplified dynamic system can be represented using

(14) $$\begin{array}{c} \ddot{x}=\frac{1}{m_{cart}}\left[-\left(k_{l}+k_{r}\right)x-c_{cart}\dot{x-\left(k_{l}-k_{r}\right)y+k_{l}r\theta_{1}}+k_{r}r\theta_{2}\right], \end{array}$$

(15) $$\begin{array}{c} \ddot{y}=\frac{1}{m_{bridge}}\left[-\left(k_{l}-k_{r}\right)x-\left(4k_{f}+k_{l}+k_{r}\right)y-c_{bridge}\dot{y}+\left(2k_{f}+k_{l}\right)r\theta_{1}-\left(2k_{f}+k_{l}\right)r\theta_{2}\right], \end{array}$$

(16) $$\begin{array}{c} {\ddot{\theta }}_{1}=\frac{1}{J_{1}}\left[k_{l}rx+\left(2k_{f}+k_{l}\right)ry-\left(k_{f}+k_{l}\right)r^{2}\theta_{1}-c_{1}{\dot{\theta }}_{1}+k_{f}r^{2}\theta_{2}+\gamma \eta k_{t}I_{1}\right]-\frac{1}{2}k_{l}{\left(-\theta_{1}r+x+y\right)}^{2}-\frac{1}{2}k_{r}{\left(\theta_{2}r-x+y\right)}^{2}, \end{array}$$

(17) $$\begin{array}{c} {\ddot{\theta }}_{2}=\frac{1}{J_{2}}\left[k_{l}rx-\left(2k_{f}+k_{l}\right)ry-\left(k_{f}+k_{r}\right)r^{2}\theta_{2}-c_{2}{\dot{\theta }}_{2}+k_{f}r^{2}\theta_{1}+\gamma \eta k_{t}I_{2}\right]. \end{array}$$

## 3. Control Design of the BULReD

### 3.1. Trajectory Tracking Control

**Figure 5:**
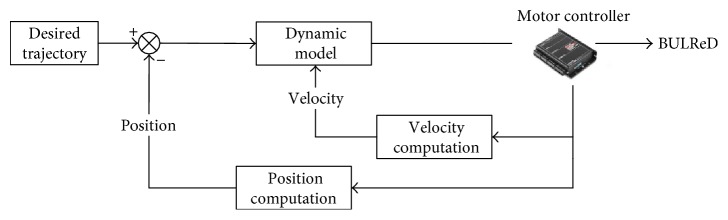
The control diagram of the BULReD for passive training.

The trajectory tracking control of the BULReD is the basis of a variety of robot-assisted rehabilitation exercises. It can be directly used for passive training on stroke patients who has week active motor ability. However, tracking desired trajectories is not only a simple but also an effective method for rehabilitation applications [35]. For passive training, patients totally follow the predefined motion trajectory of the BULReD. The proposed control techniques are presented in Figure 5.

### 3.2. Interaction Control

**Figure 6:**
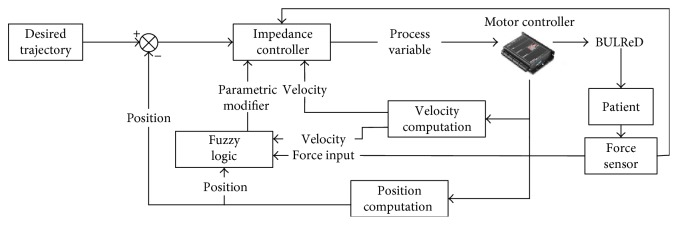
Control diagram transmission for assistive rehabilitation.

To ensure active participation from patients during the robotic training, and hence enhance the training efficacy, a fuzzy adaptive-based variable impedance (FABVI) controller has been given to modify parameters of stiffness and damping according to desired force with detected user's force. The proposed control techniques are presented in Figure 6. Considering the impaired limb, the desired impedance property between robot and impaired limb can be given in

(18) $$\begin{array}{c} M\ddot{X}+B\left({\dot{X}}_{d}-\dot{X}\right)+K\left(X_{d}-X\right)=f-f_{e}, \end{array}$$

where *M*, *B*, and *K* are variable inertia, damping, and stiffness matrix, respectively; *X*_*d*_ is the 2×1 vector of the desired position of device end-effector; and *X* is the 2×1 vector of the current location;$\;{\dot{X}}_{d}$,$\;\dot{X,}\;$and$\;\ddot{X\;}$are first and second derivatives individually; *f* is the end-effector input force; and  *f*_*e*_ represents limb applying force on the device. The fuzzy inference rules are shown in Table 2.

The fuzzy subsets are defined as follows:

(19) $$\begin{array}{c} e_{p}=X_{d}-X=\left\{NB,NM,NS,O,PS,PM,PB\right\}, \end{array}$$

(20) $$\begin{array}{c} e_{v}={\dot{X}}_{d}-\dot{X}=\left\{NB,NM,NS,O,PS,PM,PB\right\}, \end{array}$$

(21) $$\begin{array}{c} e_{f}=f-f_{e}=\left\{NB,NM,NS,O,PS,PM,PB\right\}. \end{array}$$

*e*_*p*_, *e*_*v*_, *e*_*f*_, and  *b*, *k* all follow Gaussian distributions.

## 4. Experiments and Results

**Figure 7:**
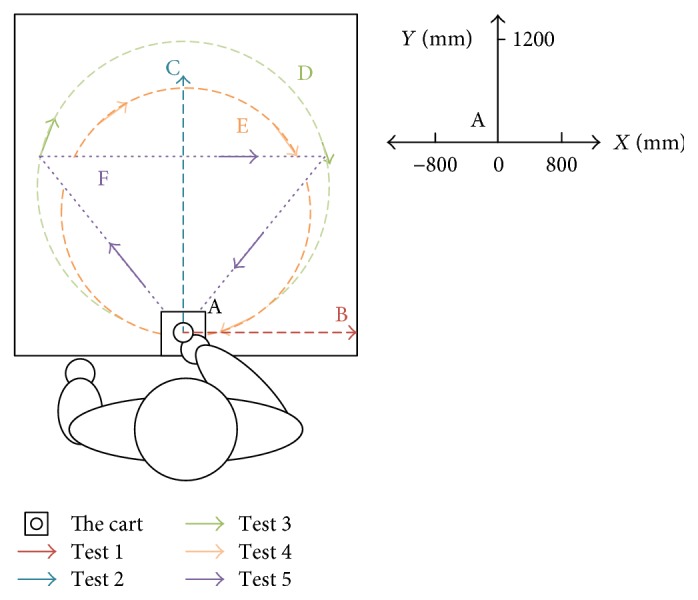
The predefined training trajectories on the BULReD. Training exercises along trajectories 1, 2, 3, 4, and 5 are denoted as tests 1, 2, 3, 4, and 5, respectively.

To preliminarily estimate the performance of the BULReD and its control system, two healthy subjects participated in the test in the lab environment. Subject A has the age of 20 years old, with 178 cm height. Subject B is a 28-year-old male, with the height of 174 cm. The max force of both pulling and pushing is 200 N. The predefined training trajectories are presented in Figure 7.

### 4.1. Trajectory Tracking Responses

**Figure 8:**
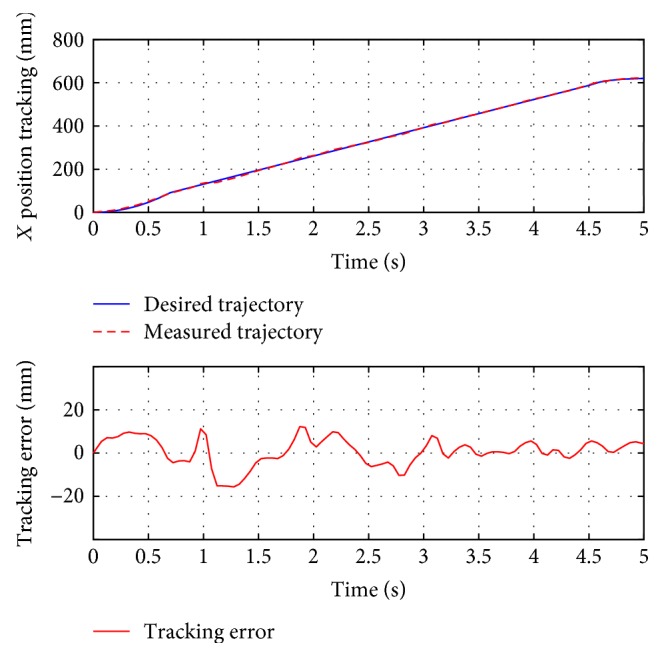
The position tracking responses of test 1.

**Figure 9:**
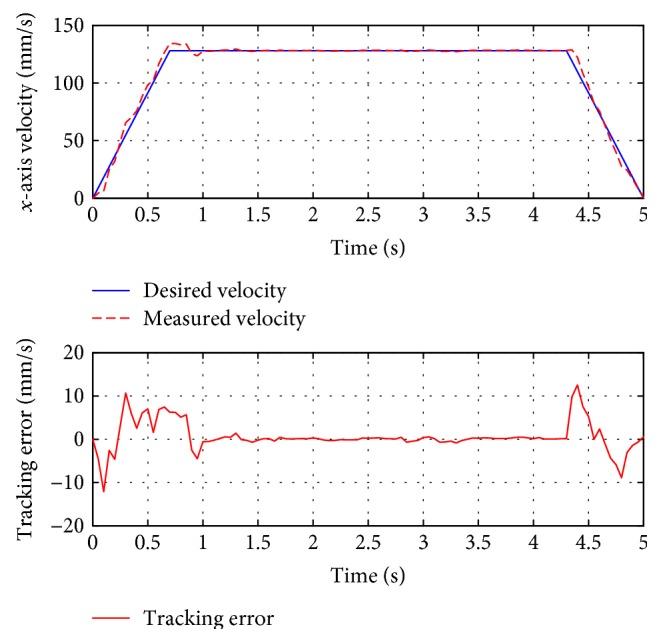
The velocity tracking responses of test 1.

**Figure 10:**
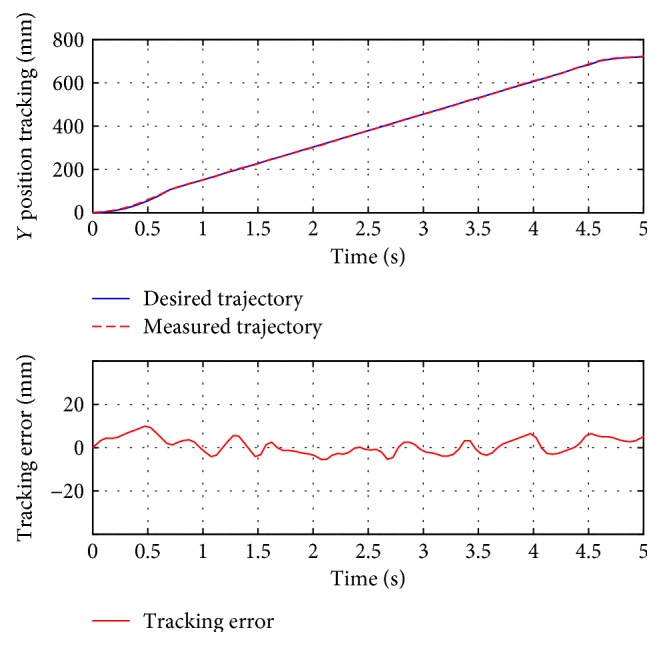
The position tracking responses of test 2.

**Figure 11:**
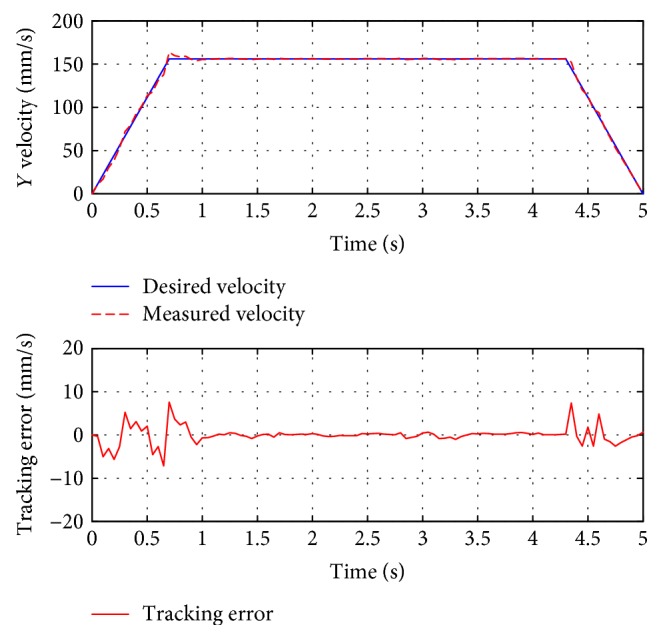
The velocity tracking responses of test 2.

**Figure 12:**
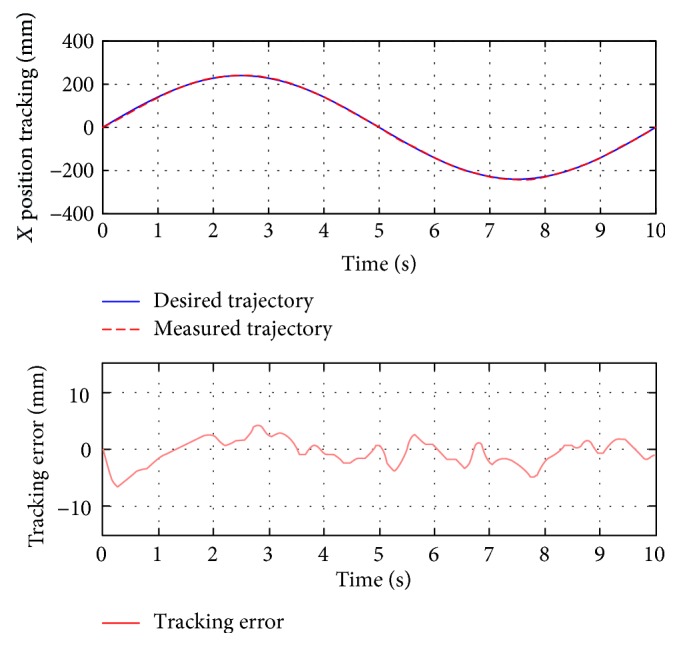
The *x*-axis position tracking responses of test 3.

**Figure 13:**
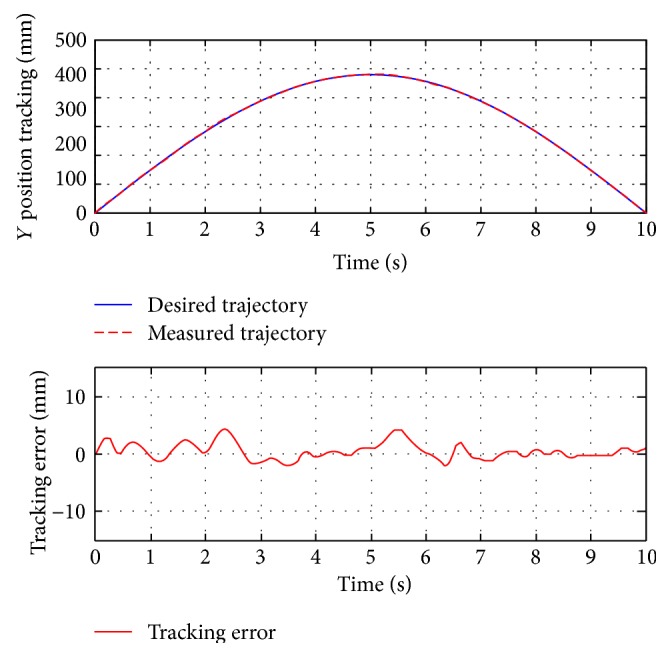
The *y*-axis position tracking responses of test 3.

For passive training, subject A was guided to finish three training trajectories (A-B, A-C, and D, also denoted as test 1, test 2, and test 3, resp.). During the whole process, the subject was encouraged to relax and did not apply intentional active force on the hand holder.  Figure 8 presents the position tracking responses of test 1, and the corresponding velocity tracking is shown in Figure 9. Both show satisfactory tracking accuracy, with the root-mean-square error (RMSE) values being 4.0558 mm for position tracking and 3.3779 mm/s for velocity tracking. Test 2 is a trajectory along the *y*-axis, and experimental results are presented in Figures 10 and 11. The achieved position tracking accuracy is an RMSE of 3.7781 mm, and for velocity tracking, it is 2.0551 mm/s. To conduct a combined motion along both *x*- and *y*-axes, test 3 was designed where the BULReD took the limbs for a circle within the workspace. The position tracking responses are presented in Figures 12 and 13 for *x*- and *y*-axes, respectively. The RMSE values are 1.8324 mm and 1.2391 mm, respectively. These statistical results are summarized in Table 3.

### 4.2. Interaction Control Responses

With respect to the FABVI controller, the initial desired impedance control parameters *M*, *B*, and *K* were chosen as 1.5, 20, and 100, respectively. Domains of the input and output variables in the fuzzy adaptive algorithm were defined as *e*_*p*_ ∈ [−1, 1], *e*_*v*_ ∈ [−1, 1], *e*_*f*_ ∈ [−1, 1], *b* ∈ [−6, 6], *k* ∈ [−6, 6]. The desired force between the BULReD and the human limbs was set at 10 N.

**Figure 14:**
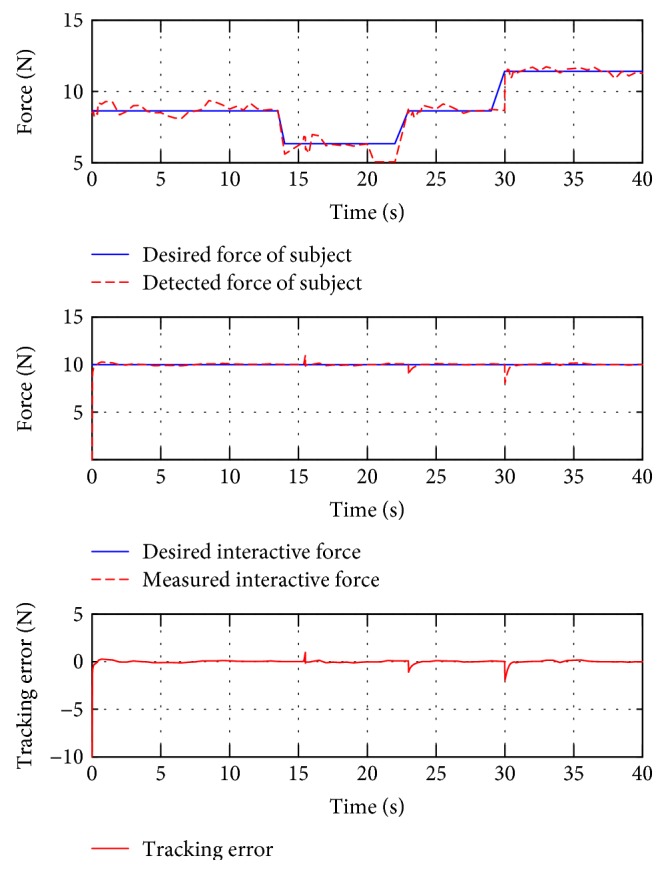
The force tracking responses of test 4.

**Figure 15:**
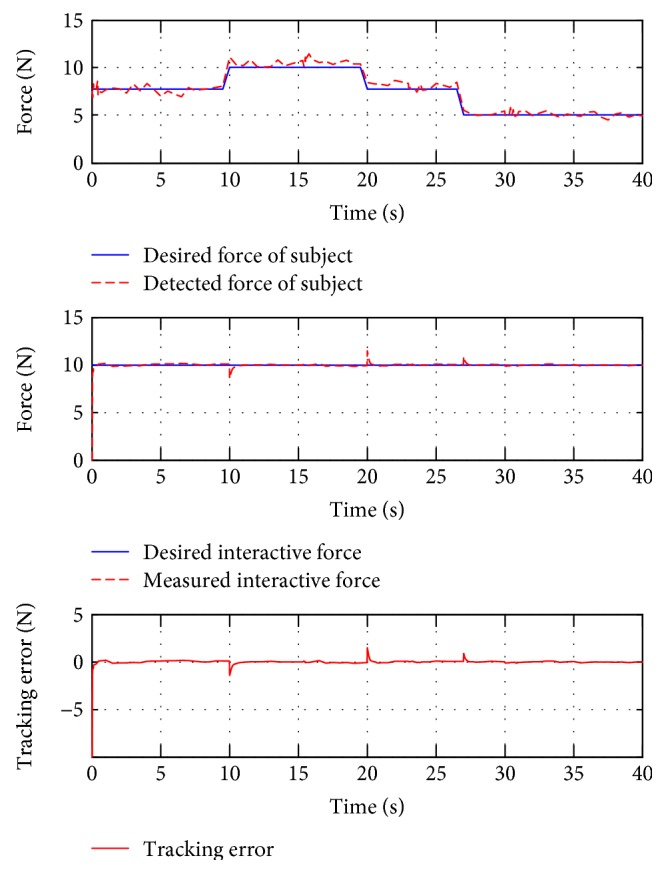
The force tracking responses of test 5.

For interactive training, subject B was guided to finish two training trajectories (E and F, also denoted as test 4 and test 5, resp.). In the case of this situation, people are demanded to use changing force to simulate stiffness or spasticity. In test 4, the subject applied an initial force 8 N, then reduced to 6 N at around the 14th second and got back to 8 N at the 23rd second, and finally changed to 11 N at the 28th second. In test 5, the initial force was also 8 N, it turned to 10 N at the 10th second, back to 8 N at the 19th second, and decreased to 5 N at the 27th second. These data are presented in the top plots of Figures 14 and 15.

Figure 14 also presents the force tracking responses of test 4 (middle plot). It is shown that the controller can achieve the reference constant force with the presence of the user's variable force applying, with the RMSE value being 1.0051 N. Similarly, Figure 15 also shows satisfactory force tracking performance, with the RMSE value at 0.9012 N. These statistical results are also included in Table 3.

## 5. Conclusions

This paper proposed a two-DOF end-effector device for bilateral upper-limb rehabilitation training. The BULReD is portable for both hospital and home environment, easy to use for therapists and patients, and safer with respect to upper-limb robotic exoskeletons. It was implemented to be able to conduct both passive and interactive trainings, based on system kinematics and dynamics, as well as the identification of real-time movement intention of human users. Preliminary results demonstrate the potential of the BULReD for clinical applications, with satisfactory position and interaction force tracking performance. Future work will focus on the clinical evaluation of the BULReD on a large sample of poststroke patients.

## Figures and Tables

**Table 1:** The qualitative description of the motion transmission of the BULReD.

Motion pattern	Motor pulley angular displacement		End-effector direction
	Motor 1	Motor 2	
1	+	·	↗
2	+	+	→
3	+	−	↑
4	−	·	↙
5	−	+	↓
6	−	−	←
7	·	+	↘
8	·	−	↖

Note: + means anticlockwise rotation, − means clockwise rotation, and · represents the motor being still.

**Table 2:** The fuzzy inference rules of *b* and *k*.

Δ*f*	NB	NM	NS	ZO	PS	PM	PB
*b*, *k*							
Δ_*v*_							
NB	NB	NB	NM	NM	NS	ZO	ZO
NM	NB	NB	NM	NS	NS	ZO	ZO
NS	NB	NM	NS	NS	ZO	PS	PS
ZO	NM	NM	NS	ZO	PS	PM	PM
PS	NM	NS	ZO	PS	PS	PM	PB
PM	ZO	ZO	PS	PS	PM	PB	PB
PB	ZO	ZO	PS	PM	PM	PB	PB

**Table 3:** Statistical results of the tracking responses of all tests.

Error type		*x*-axis position (mm)	*y*-axis position (mm)	*x*-axis velocity (mm/s)	*y*-axis velocity (mm/s)	Force (N)
RMSE						
Mode						
Passive mode	Test 1	4.0558	N\A	3.3779	N\A	N\A
	Test 2	N\A	3.7781	N\A	2.0551	N\A
	Test 3	1.8324	1.2391	N\A	N\A	N\A

Assistive mode	Test 4	N\A	N\A	N\A	N\A	1.0051
	Test 5	N\A	N\A	N\A	N\A	0.9012

RMSE: root-mean-square error; N\A: not available.
